# The responsiveness of sensibility and strength tests in patients undergoing carpal tunnel decompression

**DOI:** 10.1186/1471-2474-12-244

**Published:** 2011-10-27

**Authors:** Christina Jerosch-Herold, Lee Shepstone, Leanne Miller, Peter Chapman

**Affiliations:** 1 Faculty of Medicine and Health Sciences, University of East Anglia, Norwich, UK; 2 Norfolk and Norwich University Hospitals NHS Foundation Trust, Norwich, UK

## Abstract

**Background:**

Several clinical measures of sensory and motor function are used alongside patient-rated questionnaires to assess outcomes of carpal tunnel decompression. However there is a lack of evidence regarding which clinical tests are most responsive to clinically important change over time.

**Methods:**

In a prospective cohort study 63 patients undergoing carpal tunnel decompression were assessed using standardised clinician-derived and patient reported outcomes before surgery, at 4 and 8 months follow up. Clinical sensory assessments included: touch threshold with monofilaments (WEST), shape-texture identification (STI™ test), static two-point discrimination (Mackinnon-Dellon Disk-Criminator) and the locognosia test. Motor assessments included: grip and tripod pinch strength using a digital grip analyser (MIE), manual muscle testing of abductor pollicis brevis and opponens pollicis using the Rotterdam Intrinsic Handheld Myometer (RIHM). The Boston Carpal Tunnel Questionnaire (BCTQ) was used as a patient rated outcome measure.

**Results:**

Relative responsiveness at 4 months was highest for the BCTQ symptom severity scale with moderate to large effects sizes (ES = -1.43) followed by the BCTQ function scale (ES = -0.71). The WEST and STI™ were the most responsive sensory tests at 4 months showing moderate effect sizes (WEST ES = 0.55, STI ES = 0.52). Grip and pinch strength had a relatively higher responsiveness compared to thenar muscle strength but effect sizes for all motor tests were very small (ES ≤0.10) or negative indicating a decline compared to baseline in some patients.

**Conclusions:**

For clinical assessment of sensibility touch threshold assessed by monofilaments (WEST) and tactile gnosis measured with the STI™ test are the most responsive tests and are recommended for future studies. The use of handheld myometry (RIHM) for manual muscle testing, despite more specifically targeting thenar muscles, was less responsive than grip or tripod pinch testing using the digital grip analyser (MIE). When assessing power and pinch strength the effect of other concomitant conditions such as degenerative joint disease on strength needs to be considered.

## Background

Carpal tunnel syndrome (CTS) is an entrapment neuropathy of the median nerve at the wrist causing numbness, tingling and pain in the palm, thumb, index and middle fingers as well as weakness of the thenar muscles. CTS is an important contributor to impaired hand function, work disability and increased dependence in adults[1]. It is estimated that one third of patients diagnosed with CTS will require surgery to decompress the carpal tunnel[2]. Surgical intervention has been found to be effective in reducing disability and improving health-related quality of life [3,4] and is cost-effective [5] in patients with moderate to severe pathology. Several systematic reviews on the effectiveness of surgical [6,7] and non-surgical treatments [8,9] have been published. However, the wide range of outcomes assessed has impeded the pooling of results for meta-analysis[10] and there is a lack of consensus on which tests should be used in trials of treatment effectiveness for CTS. In a qualitative study on what patients consider important criteria for judging the success of surgery [11] the following outcomes were identified: symptom resolution, specifically relief of hand numbness, hand pain and nocturnal waking, improved muscle function, return to work and resumption of everyday activities. A range of patient-rated outcome measures and clinically derived assessments are available to measure these domains and a combination of both patient-reported and clinician-derived outcomes is advocated ensuring that patients' perspectives on their health status are included in evaluations of effectiveness.

The Boston Carpal Tunnel Questionnaire (BCTQ) [12] is a disease-specific, patient-reported questionnaire which has extensive research underpinning its validity, reliability and responsiveness in patients with CTS [13]. It has been widely used as a primary outcome measure in trials and has shown superior responsiveness compared to other region-specific or generic patient-rated questionnaires such as the DASH or SF-36 [14,15]. However the answer to the question of which physical measures of hand sensibility and hand strength should be used in clinical trials in CTS has continued to elude us. As a result clinicians and researchers continue to use different tests to assess sensibility and strength making comparison across centres difficult. Moreover the use of multiple tests of sensory and motor function may unnecessarily duplicate information whilst increasing assessor burden.

Several tests of sensibility are available and studies on their validity, reliability and responsiveness in peripheral nerve injuries have been published [16], but little is known about their relative responsiveness in patients undergoing surgery for carpal tunnel syndrome. A systematic review of the outcomes assessed in 28 randomised controlled trials comparing open with endoscopic carpal tunnel release [17] found that sensory function was assessed by a range of clinical performance tests in 15/28 studies and motor function in 24/28 studies. A subsequent statistical review of those trials assessing grip, pinch and manual muscle strength [18] found that a wide range of strength tests were used. Although grip strength assessed by hydraulic dynamometry was most commonly used it was not the most responsive indicator of change. This may in part be explained by the fact that power grip does not specifically target the thenar muscles and may also be affected in the short-term by pain and tenderness over the surgical scar. The authors recommended further investigation into different methods of assessing power, pinch grip and manual muscle testing including the use of handheld myometry for manual muscle testing of thenar muscles and over a longer follow-up period than 12 weeks. This prospective observational study was designed to investigate the relative responsiveness of several clinically derived tests of motor and sensory function in patients undergoing carpal tunnel decompression over a follow-up period of 8 months after surgery. The tests which best capture change can then be recommended as part of a core set of outcome measures for use in clinical practice and future trials.

## Methods

We conducted a longitudinal cohort study using repeated measures of clinician derived and patient-reported outcomes. The study received full approval from the Norfolk Research Ethics Committee (Ref 09/H0310/2) and the local NHS Trust Research and Development Department. All participants gave written informed consent.

Patients were recruited from a single centre, the Norfolk and Norwich University Hospital covering the operating lists of four consultant orthopaedic or plastic surgeons. Patients were identified from the Day Procedure Unit's surgical waiting lists, which is made up of patients who have been referred either directly by their general practitioner or seen by an orthopaedic or plastic surgeon and listed for carpal tunnel decompression. CTS was diagnosed by signs, symptoms and clinical history and nerve conduction studies (NCS) were only carried out in those patients in whom the diagnosis by clinical presentation was uncertain. As not all patients undergo NCS and due to ethical constraints of accessing such data it was not possible to classify CTS severity.

Administrative staff at the Day Procedure Unit were asked to mail participant information sheets, consent forms and pre-assessment questionnaires with a self addressed envelope to all patients listed for carpal tunnel surgery between April 2009 and April 2010. Patients were given 1 week in which to decide whether to take part and return their signed consent form and screening questionnaire by mail. Inclusion criteria were a confirmed diagnosis of CTS through a clear clinical history with or without neurophysiological examination, listed for surgical decompression with a date of surgery at least 2 weeks away, aged 18 or over and able to give fully informed consent.

Patients returning a signed consent form were invited to attend for their presurgical assessment at the Clinical Trials Unit at the University of East Anglia.

Clinician derived measures of sensory and motor function which have been standardised on populations with peripheral nerve trauma and/or compression were used. Clinical assessments were carried out by two qualified occupational therapists specialising in hand therapy and experienced in their use. A standardised protocol was followed for each test. The order of testing was randomised to control for possible order effects. Four sensory function tests were selected based on a review of evidence regarding their validity and reliability in peripheral nerve injuries (Jerosch-Herold 2003).

1) Touch threshold was measured using the Weinstein Enhanced Sensory Test (WEST) (Bioinstruments, Connecticut, USA) which has been demonstrated to have high validity and excellent inter-and intra-tester reliability [19]. The WEST monofilaments have improved tip geometry reducing slippage [20]and are supplied with guaranteed calibration. An ascending method of threshold testing was used starting with the lightest filament and randomly interspersing 3 stimuli with 2 'shams'. Detection of at least 1 out of 3 stimuli was used to determine the lowest threshold and recorded on an ordinal scale as follows: 0.07 gm = 4: 0.2 gm = 3: 2.0 gm = 2; 4.0 gm = 1; 200 gm = 0; The tip of the thumb and index finger were tested and a mean score calculated.

2) Static two-point discrimination (2PD) was measured using the Dellon-Mackinnon Disk-Criminator ™(AliMed, MA, USA) following Moberg's protocol [21]. Starting with a calliper distance of 5 mm a few random applications of 1 or 2 points was used to determine if patients could discriminate correctly then increasing or decreasing the distance depending on responses. The final threshold was determined as the smallest distance at which at least 7 out of 10 applications were correctly identified. The tip of the thumb and index finger were tested and a mean value calculated.

3) Locognosia was assessed using a standardised area localisation test [22]. Using a hand map in which the fingertips are divided into four quadrants and consecutively numbered, patients were asked to identify the exact quadrant in which they felt a stimulus using the heaviest monofilament on the WEST (200 gms). Each zone is stimulated twice in a pre-randomised order and 1 point is given for each correctly identified digit and quadrant, respectively. Only the median nerve innervated area was tested with a maximum possible score of 56 points.

4) Tactile gnosis was assessed using the Shape Texture Identification (STI™) Test (Ossur, Sweden) according to a standardised protocol [23]. Using three shapes and three textures of decreasing size fixed onto disks, patients are required to use their index fingertip to correctly identify each shape and texture. A maximum of 6 points can be scored.

The choice of motor tests was based on a systematic review of clinical trials and statistical review of responsiveness previously undertaken [18].

Functional grip and tip pinch strength were assessed using the MIE digital grip analyser (MIE Medical Research Ltd, Leeds, UK). This instrument has lightweight padded handles attached to a strain gauge tension dynamometer and was chosen for its ability to register even very weak grip and greater handle comfort compared to the hydraulic dynamometer which requires a visual reading from a scale and has been shown to have flooring effects [24]. A standardised protocol was used to measure power grip and tip pinch using the positioning recommended by the Clinical Assessment Recommendations of the American Society of and Therapists [25]. The mean of three trials was recorded.

Individual muscle testing of Opponens Pollicis (OP) and Abductor Pollicis Brevis (APB) was performed using the Rotterdam Intrinsic Handheld Myometer (RIHM)(Erasmus Medical Centre, Rotterdam) The RIHM is a digital handheld myometer which allows individual muscle strength to be measured and quantified in Newtons of force. It has not been used with CTS patients, however it is validated for use in peripheral nerve injuries [26,27] and has been shown to have excellent test-retest reliability. It is much more sensitive to small changes compared to the ordinal Oxford scale for manual muscle testing but a grade 3 or more must be achieved on the Oxford scale in order to complete the RIHM testing. A standardised protocol was followed and the average of three trials was recorded. Prior to testing motor function all patients were asked whether they had pain on gripping or pinching at the base of their thumb and this was recorded.

Data on clinical presentation of CTS were collected using the clinical history questionnaire developed by Bland [28] which was incorporated into a screening questionnaire sent as part of the initial invitation. It has been shown to have an overall sensitivity of 79% when compared to nerve conduction results as gold standard [28].

Patient rated symptom severity and functional status were assessed using the disease-specific Boston Carpal Tunnel Questionnaire [29]. It is made up of two scales - the symptom severity scale (SSS) which contains 11 questions and the functional status scale (FSS) which has 8 questions. Patients were asked to rate the severity or difficulty from 1 to 5 and an average total score calculated for each subscale where a higher score indicates worse symptoms and function, respectively. Patients received the questionnaire by mail and were asked to bring it completed to their baseline assessment.

The same battery of tests was used for all follow-up assessments. Additionally we used a subjective global rating scale at the 4 and 8 month follow-up assessments. Participants were asked, prior to completing their objective assessments, whether they felt that overall their hand had improved, stayed the same or deteriorated since surgery. This was used as an external criterion to determine clinically important change and test for differences between those improved and those who remained the same or got worse. Such anchor-based approaches are considered important in externally validating whether the change observed in outcome measures relates to the patient's perceived improvement and is of clinical importance [30].

### Statistical methods

Descriptive statistics were used to summarise the characteristics of the cohort and the outcomes at baseline, 4 and 8 months. Responsiveness for each outcome variable over time was quantified using the Effect Size (mean change divided by the baseline standard deviation) and the Standardised Response Mean (mean change divided by the standard deviation of change) (SRM). An effect size of <0.3 is considered small, 0.5 is moderate and >0.8 large [31].

The change in outcome was also considered by Patient Global Assessment. A two-sample *t*-test (with 95% confidence interval) was used to test for a mean difference in change between those reporting an improvement and those not.

## Results

**Figure 1:**
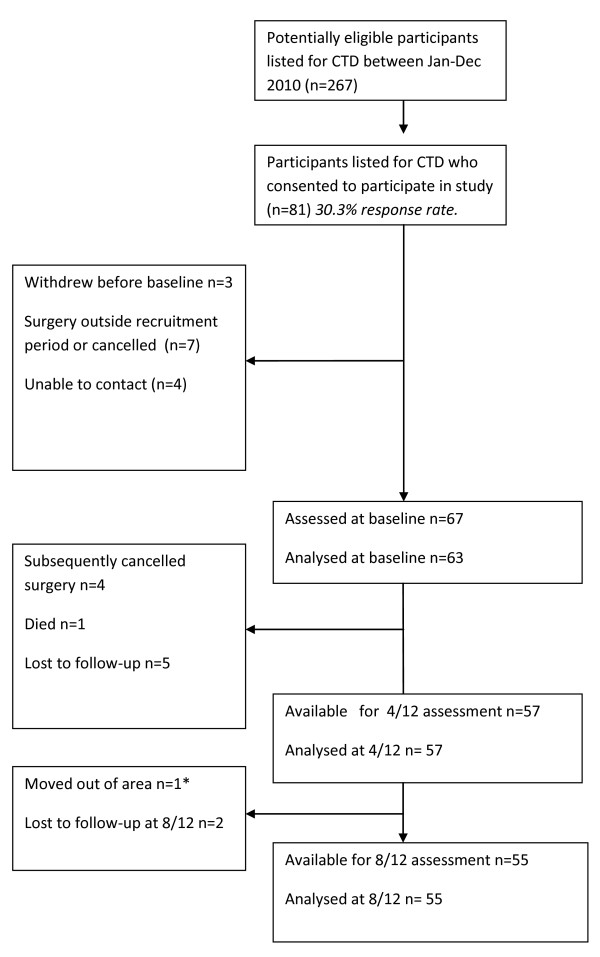
**Flowchart of patients invited, recruited and follow-up**. *Returned BCTQ by post but no other measures taken.

Between April 2009 and April 2010 a total of 267 patients listed for carpal tunnel decompression were invited to participate in the study. 81 (30.3%) patients consented of whom 67 attended a baseline assessment. Four patients subsequently postponed or cancelled their surgery thus becoming ineligible for the study and these were excluded from the baseline analysis (see Figure 1).

Table 1 summarises the sociodemographic and clinical characteristics of the 63 patients assessed at baseline. There was an almost equal distribution of gender and the mean age of the sample was 60.4 years. 38 patients had idiopathic CTS.

**Table 1:** Baseline Characteristics (N = 63)

Gender	Male	31	Relief by Shaking?	Yes	44
	Female	32		No	19
Age	Mean (SD)	60.4 (13.5)	Relief by splinting?	Yes	14
	range	30 to 91			
				No	11
Side Operated	Left	24		No splint given	38
	Right	28	Length of symptoms?		
				0-3 months	1
	Both	11		4-6 months	8
				7-12 months	9
Previous Operation	Yes	4		>12 months	45
	No	59			
			When are symptoms worse?	at Night	50
Hand Dominance	Left	3		in the morning	33
	Right	58		while working	42
	Ambidextrous	2		while driving	32
Worse Side	Left	22	Previous Stroke		4
	Right	26	Diabetic		12
	Both	15	On Dialysis		0
			Pregnant		0
Worse Finger	thumb	40	Arthritis		13
	index	52	Previous Trauma		2
	middle	60			
	ring	36			
	little	16			

**Figure 2:**
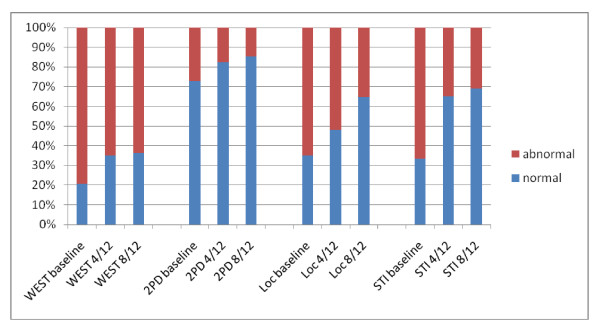
**Percentage of patients with normal and abnormal results for each sensory test at baseline, 4 and 8 months after surgery**. WEST = Weinstein Enhanced Sensory Test (abnormal <grade 4), 2PD = two-point discrimination (abnormal ≥5 mm), Loc = locognosia test (abnormal ≤48 points), STI = Shape Texture Identification test (abnormal ≤ 5 points).

Summary statistics for the clinical sensory and motor tests and self-reported symptom and function scale at baseline, 4 months and 8 months follow-up are presented in table 2. The proportion of patients scoring normal and below normal results for each of the 4 sensory tests and at each timepoint is presented in Figure 2. Two-point discrimination was normal in more than 70% of patients pre-operatively whereas touch threshold, locognosia and tactile gnosis were the tests showing the largest proportion of patients with pre-operative sensory deficits. By 8 months tactile gnosis was still below normal for 30% of patients.

**Table 2:** Outcome measures at baseline and follow-up

Outcome variables	Baseline Mean (SD) [range] N = 63	4 Months Mean (SD) [range] N = 57	8 Months Mean (SD) [range] N = 55
WEST (0-4, 4 = normal)	2.77 (0.93) [0 to 4]	3.25 (0.76) [1 to 4]	3.37 (0.64) [1.5 to 4]
2-PD (2 to >15 mm)	4.32 (3.00) [2 to >15]	3.75 (3.07) [2 to >15]	3.45 (2.82) [2 to >15]
Locognosia (0 to 56)	45.6 (6.75) [21 to 55]	47.9 (5.85) [30 to 56]	48.8 (6.80) [25 to 56]
STI Test (0 to 6, 6 = normal)	4.32 (1.74) [0 to 6]	5.18 (1.60) [0 to 6]	5.31 (1.39) [0 to 6]
			
Grip (Newtons of force)	223.0 (92.4) [38.7 to 452.3]	236.2 (84.2) [98.3 to 465.3]	235.5 (91.0) [68 to 460.1]
Pinch (Newtons of force)	59.7 (27.0) [8.33 to 116]	63.9 (22.5) [26 to 112.3]	63.3 (23.5) [21.7 to 129.3]
RIHM Abduction (Newtons)	34.3 (11.7) [7.3 to 60]	32.5 (13.2) [0 to 59.3]	30.9 (12.4) [7.5 to 54.4]
RIHM Opposition (Newtons)	31.6 (12.0) [9.3 to 61.1]	31.9 (15.3) [0 to 76]	32.7 (14.2) [6.7 to 71.3]
			
BCTQ Symptoms (1 to 5, 1 = no symptoms)	2.82 (0.78)	1.71 (0.59)	1.63 (0.69)
BCTQ Function (1 to 5, 1 no difficulty)	2.19 (0.79)	1.64 (0.59)	1.63 (0.79)
			
Global rating of Improvement :	---	47/57 (82%)	49/56 (88%)

Table 3 presents the relative responsiveness of the four sensory and four motor tests and the symptom and function subscale of the BCTQ. ES and SRM for 2PD were negative as a reduction in threshold, measured in mm, indicates an improvement. For abduction and opposition measured by RIHM a negative ES or SRM indicates a decline in strength.

**Table 3:** Change from Baseline

	4 Month Change Mean (SD) N = 57	ES	SRM	8 Month Change Mean (SD) N = 55	ES	SRM
West	0.51 (0.86)	0.55	0.59	0.68 (0.81)	0.73	0.84
2-PD	-0.65 (1.13)	- 0.22	- 0.57	- 0.99 (1.93)	- 0.33	- 0.51
Locognosia	1.95 (5.34)	0.29	0.37	2.78 (6.69)	0.41	0.42
STI Test	0.93 (1.41)	0.53	0.66	1.11 (1.58)	0.64	0.70
						
Grip	7.55 (52.9)	0.08	0.14	9.13 (61.3)	0.10	0.15
Pinch	2.01 (14.4)	0.07	0.14	1.95 (16.0)	0.07	0.12
RHIM Ab	-2.74 (10.9)	- 0.23	- 0.25	- 4.38 (8.29)	- 0.37	- 0.53
RHIM Opp	0.81 (16.7)	0.07	0.05	- 0.02 (14.1)	- 0.00	- 0.00
						
BCTQ Symp	-1.12 (0.88)	- 1.43	- 1.27	- 1.18 (0.92)	- 1.51	- 1.28
BCTQ func	-0.56 (0.71)	- 0.71	- 0.79	- 0.54 (0.72)	- 0.68	- 0.75

ES = effect size (mean of change÷ standard deviation of baseline), SRM = standardised response mean (mean of change ÷ SD of change)

A negative ES or SRM denotes a decrease in score from baseline. In 2PD (mm distance) and the BCTQ subscales (scored 1 to 5) a decrease or negative value indicates an improvement, whereas for all four motor tests a negative value indicates a decline in strength (Newtons).

Moderate effect sizes were observed for the WEST and STI tests at 4 months and 8. All four motor tests had either small or negative effect sizes. The BCTQ SSS was the most responsive test with a large ES, followed by the FSS over a 4 months follow-up.

Table 4 presents the change from baseline to 4 and 8 months for those patients who had self-reported an improvement and those who had remained the same or become worse. Ten patients considered that they had either not improved or got worse by 4 months and two patients went on to have revision surgery. There were statistically significant differences between the improved and not improved groups at 4 months in the BCTQ SSS and FSS. A highly significant difference was also seen between these groups in grip at 4 months with the not improved group showing a mean decline in strength of -17.33 Newtons from baseline. No significant differences were observed between improved and not improved for any outcomes by 8 months.

**Table 4:** Change from Baseline by Patient Global Assessment

	Not Improved Mean (SD)	Improved Mean (SD)	Difference	p-value	**95% C.I**.
4 Months					
West	0.55 (1.23)	0.50 (0.77)	0.05	0.904	-0.55 to 0.65
2-PD*	- 0.10 (1.31)	- 0.77 (1.07)	0.67	0.158	-0.11 to 1.44
Locognosia	1.30 (7.17)	2.09 (4.95)	- 0.79	0.747	-4.55 to 2.98
STI Test	0.20 (2.15)	1.09 (1.18)	- 0.89	0.235	-1.85 to 0.08
Grip	-17.33 (21.2)	12.96 (56.2)	- 30.30	**0.007**	-66.70 to -6.11
Pinch	-5.30 (13.4)	3.56 (14.3)	- 8.86	0.082	-18.74 to 1.01
RIHM Ab	-2.36 (10.9)	-2.79 (11.0)	0.44	0.924	-8.51 to 9.38
RIHM Opp	1.12 (19.2)	0.75 (16.4)	0.37	0.958	-11.94 to 12.68
BCTQ Symp*	- 0.51 (0.80)	- 1.25 (0.85)	0.74	**0.021**	0.15 to 1.32
BCTQ func*	0.08 (0.69)	- 0.69 (0.65)	0.77	**0.007**	0.31 to 1.22
					
8 Months					
West	1.07 (1.06)	0.63 (0.76)	0.45	0.318	-0.20 to 1.10
2-PD*	-0.50 (2.77)	-1.06 (1.81)	0.56	0.619	-1.01 to 2.14
Locognosia	-0.33 (8.62)	3.17 (6.41)	-3.50	0.375	-0.49 to 3.63
STI Test	1.57 (2.23)	1.04 (1.49)	0.53	0.562	-0.76 to 1.82
Grip	24.14 (84.2)	6.90 (58.0)	17.24	0.617	-32.85 to 67.33
Pinch	2.09 (19.9)	1.93 (15.6)	0.16	0.984	-12.96 to 13.29
RIHM Ab	-5.55 (3.88)	-4.26 (8.66)	-1.29	0.565	-9.22 to 6.64
RIHM Opp	2.16 (23.7)	-0.31 (12.8)	2.47	0.811	-9.98 to 14.92
BCTQ Symp*	-1.14 (1.43)	-1.18 (0.85)	0.04	0.944	-0.71 to 0.79
BCTQ func*	-0.08 (0.87)	-0.61 (0.68)	0.52	0.170	-0.04 to 1.09

*for these measures a negative mean value indicates an improvement; for all other measures improvement is indicated by a positive mean value and decline by a negative mean value

## Discussion

This study is the first to present a comparison of the relative responsiveness of several clinical sensory and motor tests in a cohort of patients who have undergone open carpal tunnel decompression and over an 8 month follow-up period.

Responsiveness statistics such as ES or SRM give a standardised score which is unit free and allows comparison between different measurement scales. ES and SRM can be interpreted using Cohen's criteria [31] whereby the larger the effect size the greater the change or response to treatment. However caution is needed in interpreting these values. Both ES and SRM use a standard deviation as denominator hence large variance in a study sample leads to a larger measure of dispersion (standard deviation) and can therefore result in small effect sizes even when the change is clinically important. Both ES and SRM are also dependent on the intervention and how much change is expected in a patient's health-status, responsiveness statistics are context-specific and there are no agreed criteria for what is a responsive measure [32]. Therefore comparing the responsiveness between tests in the same group of patients all undergoing surgery for CTS is more appropriate than between studies, as it is based on the interpretation of the relative magnitude of ES or SRM rather than the absolute values.

Of the four clinical sensory tests the most responsive tests at 4 months from baseline were touch threshold assessed by WEST (ES = 0.55, SRM = 0.59) and tactile gnosis assessed with the STI test (ES = 0.53, SRM = 0.66) which showed moderate effect sizes. This was followed by locognosia (ES = 0.29, SRM = 0.37) and 2PD (ES = -0.22, SRM = -0.57) with small effect sizes. The responsiveness statistics increased slightly by 8 months indicating that sensibility continued to improve between 4 and 8 months but also remained abnormal for touch threshold, locognosia and tactile gnosis for more than 30% of patients. Notable is that 2PD was normal (<5 mm) in 46 of 63 patients at baseline (mean at baseline = 4.3 mm) increasing only by 1 patient at 4 and 8 months. A number of authors have reported that 2PD often remains normal in CTS when other sensibility indices show abnormal results [33] and that touch threshold is more responsive than 2PD [34]. The responsiveness for sensibility tests in CTS has been reported for touch threshold [14]. In a sample of 22 patients followed for 3 months after surgery a small change was observed (SRM = -0.30) in touch threshold assessed by monofilaments. Appleby et al [35] investigated change over 3 months after surgery in 29 patients. Although they did not report responsiveness statistics these could be calculated from the data presented. Touch threshold had a moderate ES (0.67). Our study is the first to include additional measures of spatial discrimination such as the locognosia test and STI test, which were moderately responsive. Both tests have been shown to have excellent discriminative validity and responsiveness in peripheral nerve injuries [22,36,23]. The results of these tests at 8 months after surgery also highlight that for a proportion of patients localisation and shape/texture identification remained impaired.

The effect sizes and standardised response means for all four motor tests were either very small, close to zero or had decreased at 4 months and 8 months. Grip and pinch strength assessed with the MIE had a relatively higher responsiveness compared to thumb abduction and opposition assessed by RIHM. Our study is the first to report on the responsiveness of the RIHM in patients with CTS. This handheld digital myometer targets the thenar muscles, specifically abduction and opposition of the thumb which are solely reliant on median nerve innervation. It combines individual manual muscle testing with myometry thus allowing strength to be measured objectively and on a continuous scale (Newtons of force) rather than subjectively grading by using the ordinal Oxford scale. We hypothesised that this method of testing thumb opposition and abduction using the RIHM would be more responsive than power or pinch strength, however our results do not support this. Power and pinch strength measured by the MIE were more responsive than the RIHM but the question remains whether both measures are required. It has been argued before that pinch strength is a more precise measure of motor impairment in CTS as it relies to a greater extent on the median nerve innervated thenar muscles, whereas in power grip weakness can be masked by the synergistic action of long flexors [18]. However the ES and SRM for grip and pinch at 4 months were very similar and by 8 months marginally higher for power grip than tip pinch. There are several plausible explanations for these findings. Power grip can decrease in the short term after surgery due to pillar pain and scar tenderness over the carpus, recovering by 8 months and even improving due to compensatory use of long flexors and the increased use of the hand in functional activities. Another factor is that other variables such as age, dominance and gender can account for large variations in strength. The variance in strength for all 4 measures in our study was large as evidenced in the wide standard deviations and as these are used as the denominator when calculating ES or SRM this can result in small responsiveness statistics. A further possibility is that other comorbidities such as degenerative joint disease, especially carpometacarpal joint osteoarthritis can significantly reduce pinch strength. In our study 17 patients (27%) reported that they had pain at the base of the thumb on forceful gripping or pinching. Furthermore, a systematic review of strength tests found that grip and pinch strength values prior to surgery were often within or close to normative values and therefore little scope remains for further improvement after surgery in some patients [18], a so called ceiling effect.

Although the responsiveness of patient-rated outcome assessed by BCTQ was not the primary focus of this study, by 4 months the BCTQ SSS showed the largest effect sizes followed by the BCTQ FSS. These findings concur with other studies that have reported large ES or SRMs for the symptom severity scale and moderate to large ES for the functional status scale at 3 months post surgery [12,14,15,37-39]. The BCTQ is a disease-specific outcome measure which addresses typical symptoms such as pain, tingling, nocturnal waking which are relieved or improved upon surgical decompression. It is therefore not surprising that this measure is more responsive than clinical tests of sensory and motor function. It is interesting though, that the responsiveness statistics for the BCTQ subscales did not increase at 8 months suggesting that the large improvement in symptom relief and functional status occurs rapidly within the first 3-4 months after surgery, whilst tactile sensibility especially spatial discrimination takes much longer to recover after surgical decompression hence the increasing effect sizes from 4 to 8 months.

The BCTQ was also the outcome measure which best differentiated between the group of patients who had improved and those unchanged or worse. The mean change at 4 months from baseline in symptom severity was -1.25 points in the improved group and -0.51 points in the same/worse group. For the functional status scale a change of -0.69 points was observed in the improved group and 0.08 points in the same/worse group. Atroshi [38] reported smaller values for minimally clinically important difference (MCID) for symptom severity (0.8 points) and for the function scale (0.5 points) using patient satisfaction as the criterion in patients undergoing surgery. Our findings also supports the notion that patients underwent 'clinically important change' as a result of surgery. They also provide an external criterion for interpreting the change scores in the clinical tests and to determine the magnitude of change which can be deemed clinically important. For example for the STI test the change from baseline in the improved group was 1.09 points as opposed to 0.20 in the same or worse group.

A potential limitation is that we did not exclude patients with other conditions. Our study sample included 4 patients who reported having had a previous stroke, 13 indicated having arthritis and 12 were diabetic. These conditions may account for weakness and/or sensory impairment from other aetiologies and which may not respond to surgery. However they are also typical of the wide range of patients undergoing surgery for CTS and therefore enhance the generalisability of our results to other surgical cohorts. A further limitation is that we were not able to objectively verify whether all patients had a median nerve pathology as not all patients have nerve conduction tests prior to surgery. Finally our sample size is relatively small although larger than other cohorts for which responsiveness of some clinical measures has been published.

## Conclusions

We conducted the first longitudinal cohort study to examine the relative responsiveness of several clinical tests of motor and sensory function. Several of the clinical measures of sensibility showed good sensitivity to change, especially the touch threshold (WEST) and tactile gnosis (STI test). We recommend the use of these two sensory tests in the assessment of outcome in future trials of interventions for CTS particularly in patients with sensory deficits. Exploring the relationship between changes in clinical sensory function and sensory parameters from nerve conduction studies would also warrant further investigation.

Clinical tests of motor function included the assessment of power and pinch grip with the MIE digital myometer and handheld myometry for thenar muscles showed very small changes with ES of 0.10 and below. Power grip was marginally more responsive than tip pinch and more responsive than abductor pollicis brevis and opponens testing with the RIHM. Despite the fact that the RIHM targets more specifically the median nerve innervated thenar muscles its low responsiveness suggests that it does not offer any benefits over the more commonly used and widely available power and pinch strength tests. Our study shows that some patients have considerable impairments in sensibility and strength before and after surgery which are not adequately captured by self-report alone and warrant the additional use of these clinical objective tests. They should be considered for inclusion as secondary outcome measures in future trials.

## Competing interests

The authors declare that they have no competing interests.

## Authors' contributions

CJH conceived the idea, CJH, LS and PC developed the protocol and LM assisted in data acquisition and data entry. LS undertook the data analysis. All authors contributed to the drafting of the manuscript and have read and approved the final version.

## Funding

The NIHR funded this work under a Career Development Fellowship (CJH).

## Pre-publication history

The pre-publication history for this paper can be accessed here:

http://www.biomedcentral.com/1471-2474/12/244/prepub

## References

[B1] Atroshi I Gummesson C Johnsson R Sprinchorn A Symptoms, disability, and quality of life in patients with carpal tunnel syndrome Journal of Hand Surgery - American Volume 1999 24 2 398 404 10.1016/s0363-5023(99)70014-6 10194028

[B2] Atroshi I Gummesson C Non-surgical treatment in carpal tunnel syndrome Lancet 2009 374 9695 1042 1044 10.1016/S0140-6736(09)61683-4 19782855

[B3] Atroshi I Larsson G-U Ornstein E Hofer M Johnsson R Ranstam J Outcomes of endoscopic surgery compared with open surgery for carpal tunnel syndrome among employed patients: randomised controlled trial.[see comment] BMJ 2006 332 7556 1473 10.1136/bmj.38863.632789.1F 16777857 PMC1482334

[B4] Jarvik JG Comstock BA Kliot M Turner JA Chan L Heagerty PJ Hollingworth W Kerrigan CL Deyo RA Surgery versus non-surgical therapy for carpal tunnel syndrome: a randomised parallel-group trial Lancet 2009 374 9695 1074 1081 10.1016/S0140-6736(09)61517-8 19782873

[B5] Korthals-de Bos IBC Gerritsen AAM van Tulder MW Rutten-van Molken MPMH Ader HJ de Vet HCW Bouter LM Surgery is more cost-effective than splinting for carpal tunnel syndrome in the Netherlands: results of an economic evaluation alongside a randomized controlled trial BMC Musculoskeletal Disorders 2006 7 86 10.1186/1471-2474-7-86 17109748 PMC1660539

[B6] Huisstede BM Randsdorp MS Coert JH Glerum S van Middelkoop M Koes BW Carpal tunnel syndrome. Part II: effectiveness of surgical treatments--a systematic review Archives of Physical Medicine & Rehabilitation 2010 91 7 1005 1024 10.1016/j.apmr.2010.03.023 20599039

[B7] Scholten R Gerritsen AAM Uitdehaag BMJ van Geldere D de Vet HCW Bouter LM Surgical treatment options for carpal tunnel syndrome The Cochrane Library 2005 2

[B8] Huisstede BM Hoogvliet P Randsdorp MS Glerum S van Middelkoop M Koes BW Carpal tunnel syndrome. Part I: effectiveness of nonsurgical treatments--a systematic review Archives of Physical Medicine & Rehabilitation 2010 91 7 981 1004 10.1016/j.apmr.2010.03.022 20599038

[B9] Mueller M Tsui D Schnurr R Biddulph-Deisroth L Hard J MacDermid JC Effectiveness of hand therapy interventions in primary management of carpal tunnel syndrome: a systematic review Journal of Hand Therapy 2004 17 2 210 228 10.1197/j.jht.2004.02.009 15162107

[B10] Gerritsen AA de Vet HC Scholten RJ van Tulder MW Bouter LM Enabling meta-analysis in systematic reviews on carpal tunnel syndrome Journal of Hand Surgery - American Volume 2002 27 5 828 832 10.1053/jhsu.2002.35074 12239672

[B11] Jerosch-Herold C Mason R Chojnowski AJ A qualitative study of the experiences and expectations of surgery in patients with carpal tunnel syndrome Journal of Hand Therapy 2008 21 1 54 61 quiz 62 10.1197/j.jht.2007.09.001 18215752

[B12] Levine DW Simmons BP Koris MJ Daltroy LH Hohl GG Fossel AH Katz JN A self-administered questionnaire for the assessment of severity of symptoms and functional status in carpal tunnel syndrome Journal of Bone & Joint Surgery - American Volume 1993 75 11 1585 1592 8245050 10.2106/00004623-199311000-00002

[B13] Leite JCdC Jerosch-Herold C Song F A systematic review of the psychometric properties of the Boston Carpal Tunnel Questionnaire BMC Musculoskeletal Disorders 2006 7 78 10.1186/1471-2474-7-78 17054773 PMC1624826

[B14] Amadio P Silverstein M Ilstrup D Schleck C Jensen L Outcome assessment for carpal tunnel surgery: the relative responsiveness of generic, arthritis-specific, disease-specific, and physical examination measures The Journal of Hand Surgery 1996 21A 338 346 10.1016/S0363-5023(96)80340-6 8724457

[B15] Greenslade JR Mehta RL Belward P Warwick D DASH and Boston Questionnaire assessment of carpal tunnel syndrome outcome: what is the responsiveness of an outcome questionnaire? J of Hand Surgery 2004 29B 2 159 164 10.1016/j.jhsb.2003.10.010 15010164

[B16] Jerosch-Herold C Assessment of sensibility after nerve injury and repair: a systematic review of evidence for validity, reliability and responsiveness of tests Journal of Hand Surgery - British Volume 2005 30 3 252 264 10.1016/j.jhsb.2004.12.006 15862365

[B17] Jerosch-Herold C Leite JCdC Song F A systematic review of outcomes assessed in randomized controlled trials of surgical interventions for carpal tunnel syndrome using the International Classification of Functioning, Disability and Health (ICF) as a reference tool BMC Musculoskeletal Disorders 2006 7 96 10.1186/1471-2474-7-96 17147807 PMC1713237

[B18] Geere J Chester R Kale S Jerosch-Herold C Power grip, pinch grip, manual muscle testing or thenar atrophy - which should be assessed as a motor outcome after carpal tunnel decompression? A systematic review BMC Musculoskeletal Disorders 2007 8 114 10.1186/1471-2474-8-114 18028538 PMC2213649

[B19] Bell-Krotoski J Tomancik E The repeatability of the Semmes-Weinstein monofilaments Journal of Hand Surgery 1987 12A 155 166 10.1016/s0363-5023(87)80189-2 3805636

[B20] Al-Quattan M Semmes-Weinstein Monofilaments versus Weinstein enhanced monofilaments: their use in the hand clinic Canadian J of Plast Surgery 1995 3 1 51 53

[B21] Moberg E The unresolved problem - how to test the functional value of hand sensibility J of Hand Therapy 1991 4 105 110

[B22] Jerosch-Herold C Rosen B Shepstone L The reliability and validity of the locognosia test after injuries to peripheral nerves in the hand Journal of Bone & Joint Surgery - British Volume 2006 88 8 1048 1052 10.1302/0301-620X.88B8.17444 16877604

[B23] Rosen B Lundborg G A new tactile gnosis instrument in sensibility testing J of Hand Therapy 1998 11 251 257 10.1016/s0894-1130(98)80020-3 9862262

[B24] Ward C Adams SA Comparative Study of the Test-Re-Test Reliability of Four Instruments to Measure Grip Strength in a Healthy Population Br J Hand Ther 2007 12 48 54

[B25] ASHT Clinical Assessment Recommendations 1992 American Society of Hand Therapists

[B26] Schreuders TAR Roebroeck ME Jaquet J-B Hovius SER Stam HJ Measuring the strength of the intrinsic muscles of the hand in patients with ulnar and median nerve injuries: reliability of the Rotterdam Intrinsic Hand Myometer (RIHM) Journal of Hand Surgery - American Volume 2004 29 2 318 324 10.1016/j.jhsa.2003.10.024 15043908

[B27] Schreuders TAR Selles RW Roebroeck ME Stam HJ Strength measurements of the intrinsic hand muscles: a review of the development and evaluation of the Rotterdam intrinsic hand myometer Journal of Hand Therapy 2006 19 4 393 401 quiz 402 10.1197/j.jht.2006.07.024 17056399

[B28] Bland JDP The value of the history in the diagnosis of carpal tunnel syndrome Journal of Hand Surgery 2000 25B 445 450 10.1054/jhsb.2000.0452 10991809

[B29] Atroshi I Breidenbach WC McCabe S Assessment of the Carpal Tunnel Outcome Instrument in Patients with Nerve-Compression Sympotms Journal of Hand Surgery - American Volume 1997 22-A 2 222 227 10.1016/S0363-5023(97)80155-4 9195418

[B30] Revicki D Hays RD Cella D Sloan J Recommended methods for determining responsiveness and minimally important differences for patient-reported outcomes Journal of Clinical Epidemiology 2008 61 2 102 109 10.1016/j.jclinepi.2007.03.012 18177782

[B31] Cohen J Statistical power for the behaviour sciences 1977 New York: Academic Press

[B32] Beaton D Bombardier C Katz J Wright J A taxonomy for responsiveness Journal of Clinical Epidemiology 2001 54 1204 1217 10.1016/S0895-4356(01)00407-3 11750189

[B33] Gelberman RH Szabo RM Williamson RV Dimick MP Sensibility testing in peripheral-nerve compression syndromes. An experimental study in humans Journal of Bone & Joint Surgery - American Volume 1983 65 5 632 638 6853569

[B34] Atroshi I Axelsson G Gummesson C Johnsson R Carpal tunnel syndrome with severe sensory deficit: endoscopic release in 18 cases Acta Orthopaedica Scandinavica 2000 71 5 484 487 10.1080/000164700317381180 11186406

[B35] Appleby MA Neville-Smith M Parrott MW Functional outcomes post carpal tunnel release: a modified replication of a previous study Journal of Hand Therapy 2009 22 3 240 248 quiz 249 10.1016/j.jht.2009.03.001 19457636

[B36] Rosen B Jerosch-Herold C Comparing the responsiveness over time of two tactile gnosis tests: two point discrimination and STI-test Br J Hand Ther 2000 5 4 114 119

[B37] Astifidis RP Koczan BJ Dubin NH Burke FD Shaw Wilgis EF Patient satisfaction with carpal tunnel surgery: self-administered questionnaires versus physical testing Hand Therapy 2009 14 2 39 45 10.1258/ht.2009.009007

[B38] Atroshi I Johnsson R Ornstein E Patient satisfaction and return to work after endoscopic carpal tunnel surgery Journal of Hand Surgery - American Volume 1998 23 1 58 65 10.1016/S0363-5023(98)80090-7 9523956

[B39] Gay RE Amadio PC Johnson JC Comparative responsiveness of the Disabilities of the Arm, Shoulder and Hand, the Carpal Tunnel Questionnaire and the SF-36 to clinical change after carpal tunnel release Journal of Hand Surgery-American Volume 2003 28A 2 250 254 10.1053/jhsu.2003.50043 12671856

